# Comprehensive analysis of diverse programmed cell death patterns in the prognosis, tumor microenvironment and drug sensitivity in hepatocellular carcinoma

**DOI:** 10.1097/MD.0000000000036239

**Published:** 2023-12-01

**Authors:** Youlin Yu, Yanglieguang Lou, Jinlong Zhu, Xiaobo Wang

**Affiliations:** a Department of Hepatobiliary Surgery, Affiliated Hospital of Shaoxing University, Shaoxing, Zhejiang, China.

**Keywords:** cell death, hepatocellular carcinoma, prognostic model, tumor immune microenvironment

## Abstract

Treatment failure in patients with liver hepatocellular carcinoma (LIHC) is primarily caused by tumor progression and therapy resistance. Tumor immunity plays a crucial role in regulating the homeostasis of cells through the process of programmed cell death (PCD). However, the expression profile and clinical significance of PCD-related genes in LIHC require further investigation. In this study, we analyzed twelve commonly observed PCD patterns to construct a prognostic model. We collected RNA-seq data, genomics, and clinical information from TCGA-LIHC and GSE14520 cohorts to validate the prognostic gene signature. We discovered 75 PCD-related differentially expressed genes (DEGs) with prognostic significance in LIHC. Using these genes, we constructed a PCD-related score (PCDscore) with an 11-gene signature through LASSO COX regression analysis. Validation in the GSE14520 cohort demonstrated that LIHC patients with high PCDscore had poorer prognoses. Unsupervised clustering based on the 11 model genes revealed 3 molecular subtypes of LIHC with distinct prognoses. By incorporating PCDscore with clinical features, we constructed a highly predictive nomogram. Additionally, PCDscore was correlated with immune checkpoint genes and immune cell infiltration. LIHC patients with high PCDscore exhibited sensitivity to common chemotherapy drugs (such as cisplatin and docetaxel). To summarize, our study developed a novel PCDscore model that comprehensively analyzed different cell death modes, providing an accurate prediction of clinical prognosis and drug sensitivity for LIHC patients.

## 1. Introduction

Liver cancer is one of the most common types of cancer worldwide, with an estimated 905,000 new cases and 830,000 deaths in 2020.^[1]^ Despite advances in treatment and early detection, the overall prognosis for liver cancer remains poor, with a 5-year survival rate of <20%.^[2]^ One of the challenges in liver cancer research is the heterogeneity of the disease, both at the molecular and cellular level. Liver cancer is known to have diverse cell-death patterns that may impact prognosis and drug sensitivity, and understanding these patterns is essential for developing effective treatments.^[3–8]^ In this study, we aim to leverage the diverse cell-death patterns in liver cancer to predict prognosis and drug sensitivity, with the ultimate goal of improving outcomes for patients with this challenging disease.

Cell death can be broadly categorized into 2 types based on the different stimuli triggering mechanism^[9]^: Accidental cell death occurs without any control or regulation; whereas programmed cell death (PCD) involves elaborate regulations and occurs through various mechanisms. PCD encompasses several forms of cell death, including apoptosis, necroptosis, ferroptosis, pyroptosis, entotic cell death, lysosome-dependent cell death, netotic cell death, parthanatos, autophagy-dependent cell death, alkaliptosis, and oxeiptosis.^[9,10]^ Cuproptosis is a recently discovered form of cell death that is triggered by copper and has been found to be strongly associated with cancer.^[11–13]^ Recent studies have identified several cell-death patterns that play important roles in liver cancer, including apoptosis, necroptosis, pyroptosis and so on.^[14]^ Apoptosis, one of the PCD, is a key mechanism for maintaining tissue homeostasis and preventing the development of cancer, which can be a target for anticancer therapy.^[15,16]^ However, liver cancer cells have been shown to develop resistance to apoptosis, which can contribute to tumor growth and metastasis.^[17–19]^ Necroptosis is a type of PCD that is triggered by various factors such as viral infections, oxidative stress, and certain chemotherapeutic drugs,^[20,21]^ which can overcome apoptosis resistance and may also activate antitumor immunity in cancer therapy.^[3]^ Necroptosis has been found to play a crucial role in the development and progression of liver cancer.^[22]^ Pyroptosis is a distinct type of cell death that is characterized by a pro-inflammatory response and differs from other forms of programmed cell death,^[23]^ which has been shown to play a role in the development and progression of liver cancer.^[24]^

Over the past few decades, it has been well established that PCD plays a crucial role in the initiation and progression of malignant tumors. Cancer cells must evade various forms of cell death to continue their development and metastasis.^[25]^ However, the specific roles of the 12 PCD patterns in LIHC have not been comprehensively elucidated. Therefore, we utilized an expression profile-based database to identify PCD-related genes that are associated with survival. Using these genes, we established a novel predictor called the PCD-related score (PCDscore), which can be used to predict both the efficacy of therapeutic interventions and the prognosis of LIHC. In summary, our investigation of the 12 different PCD patterns in liver cancer aims to improve our understanding of the disease and pave the way for more personalized and effective treatments for LIHC patients.

## 2. Materials and methods

### 2.1. Data collection

We obtained key regulatory genes related to programmed cell death (PCD) patterns from a previous article (Supplementary Table 1, http://links.lww.com/MD/K816).^[26]^ We then accessed RNA sequencing (RNA-seq) data of 371 liver hepatocellular carcinoma (LIHC) patients and 50 normal samples from the University of California Santa Cruz database (https://genome.ucsc.edu). This data was normalized and log2 converted to RNA expression profiles FPKM, with ensemble ids manually converted to gene symbols. Additionally, clinical data was collected. We retrieved gene expression profiles and clinical information from GSE14520-GPL3921 with 220 liver non-tumor tissues and 225 HCC tissues from the Gene Expression Omnibus database (https://www.ncbi.nlm.nih.gov/geo/). Batch effects were removed using the sva package and the data was normalized using the limma R package.^[27]^

### 2.2. Identification of PCD-related gene expression and variation levels

We obtained FPKM data from 371 LIHC patients and 50 normal tissues from the TCGA-LIHC cohort. Differential expression analysis was performed using the “limma” package with the threshold of adjusted *P* value < .05 and |log2FC| > 1.^[27]^ We utilized the Sangerbox online analysis tool (http://vip.sangerbox.com/home.html) to investigate somatic mutation information in LIHC patients that may be masked.

### 2.3. Developing the PCD-related gene signature

Firstly, univariate Cox regression was applied to evaluate the impact of these PCD-related genes on the survival of LIHC patients. Then, we utilized the LASSO Cox regression method to shrink the candidate genes and select the most suitable genes to construct the signature. We determined the “lambda. min” value using the “glmnet” R package. Eventually, the PCDscore of each patient was computed by the formula: PCDscore = n ∑ Ci*Ei, where Ci represents the risk coefficient and Ei refers to the expression level of each gene. Based on the median PCDscore, we classified LIHC patients into low- and high-PCDscore groups. Principal component analysis (PCA) was performed using the “stats” package, and the correlation between overall survival (OS), disease-free interval (DFI), and progression-free interval (PFI) time and PCDscore was investigated using the “survival” and “survminer” packages for Kaplan–Meier analysis.

### 2.4. Functional enrichment analysis

To identify possible biological pathways based on the prognostic DEGs, we utilized the “clusterProfiler” R package.^[28]^ For Gene Set Enrichment Analysis (GSEA), we obtained version 3.0 of the GSEA software from the GSEA website (http://software.broadinstitute.org/gsea/index.jsp).^[29]^ To evaluate the relevant pathways and molecular mechanisms, we divided samples into 2 groups based on the median PCDscore and downloaded the h.all.v7.4.symbols.gmt subset from the Molecular Signatures Database (http://www.gsea-msigdb.org/gsea/downloads.jsp). For Gene Set Variation Analysis (GSVA), we calculated the enrichment scores of each sample in gene sets using the “GSVA” R package.^[30]^ We pre-defined gene ranks using the method proposed by Hänzelmann et al^[30]^ with gene expression profiles and downloaded the c2.cp.kegg.v7.4.symbols.gmt and h.all.v7.4.symbols.gmt subsets from the Molecular Signatures Database to assess relevant pathways and molecular mechanisms.

### 2.5. Identification of unidentified LIHC subtypes based on PCD-related gene signature

To identify the unidentified subtypes of LIHC based on PCD-related gene signature, we performed consensus clustering using the “ConsensusClusterPlus” package.^[31]^ We selected “maxK” as 9, “clusterAlg” as “km,” and “distance” as “pearson.”

### 2.6. Independent prognostic of the PCDscore

To evaluate the independent prognostic value of PCDscore, we collected clinical information (including age, gender, T, N, M, grade, and stage) of LIHC patients from the TCGA cohort, and performed both univariate and multivariable Cox regression analyses in conjunction with PCDscore.

### 2.7. Nomogram development to predict the prognosis of LIHC patients

We established a nomogram using the clinical characteristics and PCDscore in the TCGA-LIHC cohort with the “rms” R package. We assessed the accuracy of the nomogram using calibration plots and decision curve analysis with the “caret” and “rmda” R packages, respectively. Additionally, we performed Receiver Operating Characteristic (ROC) analyses using the “timeROC” R package.

### 2.8. Tumor microenvironment analysis

We utilized the “GSVA” R package to calculate the activity of immune cells, functions, and pathways for each LIHC sample through a single-sample gene set enrichment analysis (ssGSEA) algorithm. In addition, we employed the “estimate” R package to evaluate the immune score and stromal score for each LIHC sample.

### 2.9. Drug sensitivity

In order to predict chemotherapy sensitivity for each LIHC sample, we employed the “pRRophetic” R package^[32]^ and the Genomics of Drug Sensitivity in Cancer database^[33]^ (https://www.cancerrxgene.org/). This involved calculating the half-maximum inhibitory concentration for common chemotherapy drugs.

### 2.10. Statistical analysis

All statistical analyses were performed using R software (version 4.1.0). Differences between 2 groups were analyzed using Student *t* test or Wilcoxon test. Kruskal-Wallis test was used to compare differences among multiple groups. Kaplan–Meier plots were used to describe survival curves and log-rank test was used to compare them. A *P* value of <.05 was considered statistically significant.

## 3. Results

### 3.1. Variant landscape of programmed cell death genes in LIHC patients

**Figure 1.**
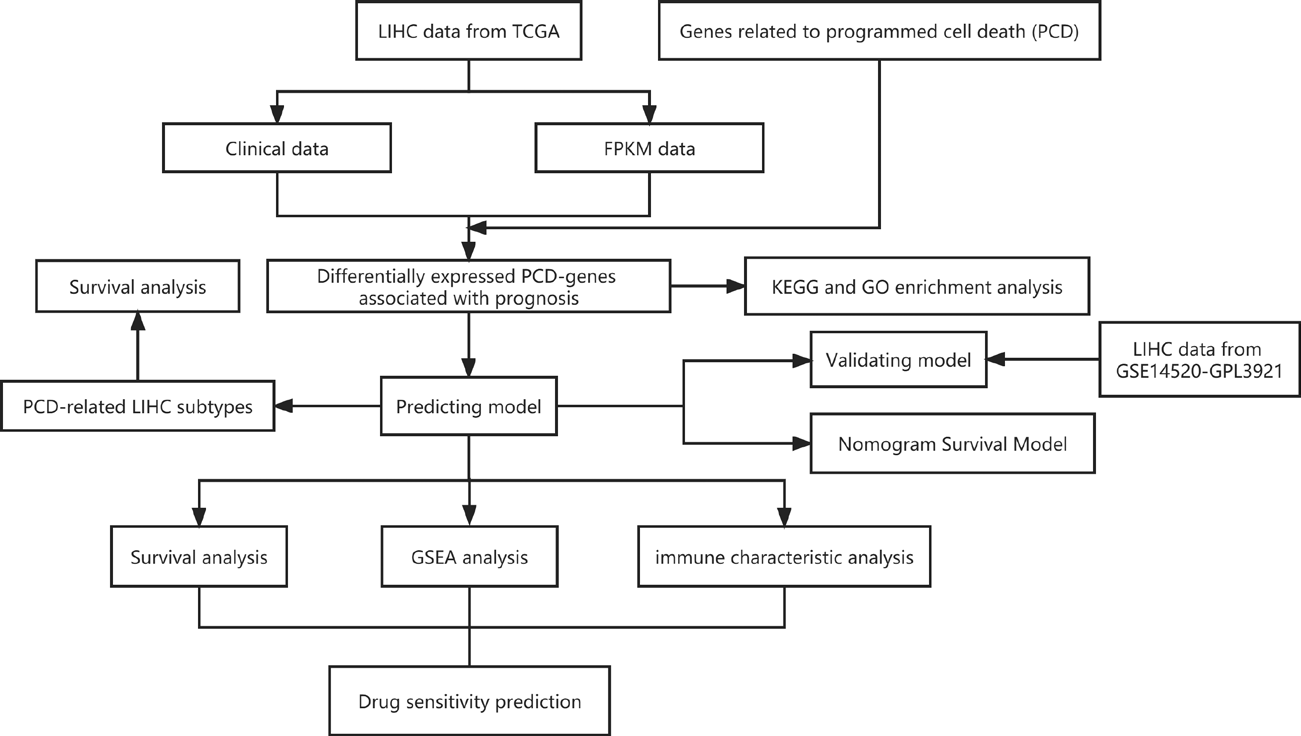
A flow diagram of the research design.

**Figure 2.**
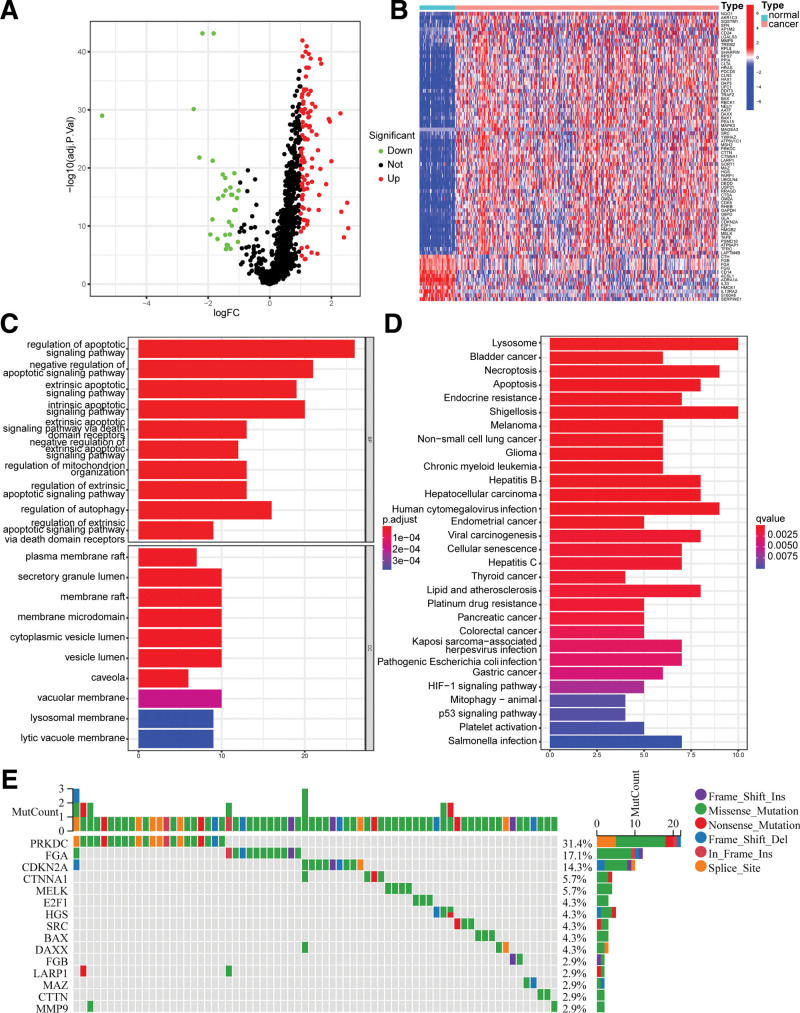
Variant landscape of PCD genes in LIHC patients. (A) Volcano plot of the prognostic PCD-related DEGs (green: down-regulated DEGs; red: up-regulated DEGs; gray: unchanged genes) (B) Heatmap of the prognostic PCD-related DEGs between LIHC and normal tissues. (C) GO enrichment analyses based on the prognostic DEGs. (D) KEGG enrichment analyses based on the prognostic DEGs. (E) An oncoplot of PCD-related genes in the TCGA-LIHC cohort. DEGs = differentially expressed genes, LIHC = hepatocellular carcinoma.

In this study, we investigated the variant landscape of programmed cell death genes in LIHC patients using the TCGA-LIHC cohort (Fig. 1). A total of 131 DEGs were identified, of which 100 were upregulated and 31 were downregulated in the LIHC group, with all DEGs listed in Supplementary Table 2, http://links.lww.com/MD/K817. Survival-related DEGs were screened using univariate Cox regression analysis, with a total of 75 genes meeting the cutoff of *P* < .05 (Supplementary Table 3, http://links.lww.com/MD/K818). A volcano plot and heatmap of the prognostic DEGs were presented in Figure 2A and B, respectively. Furthermore, KEGG and GO enrichment analysis revealed that these prognostic DEGs were involved in various biological pathways, including necroptosis, apoptosis, P53, platinum drug resistance, and HIF-1 signaling pathways (Fig. 2C and D). In addition, we evaluated the variation in PCD-related genes in LIHC patients from the TCGA cohort and identified the top 15 mutations of PCD-related genes. The PRKDC gene showed the highest mutation frequency at 31.4%, while the others ranged from 2.9% to 17.1% (Fig. 2E).

### 3.2. Construction of a prognostic gene signature for LIHC patients

**Figure 3.**
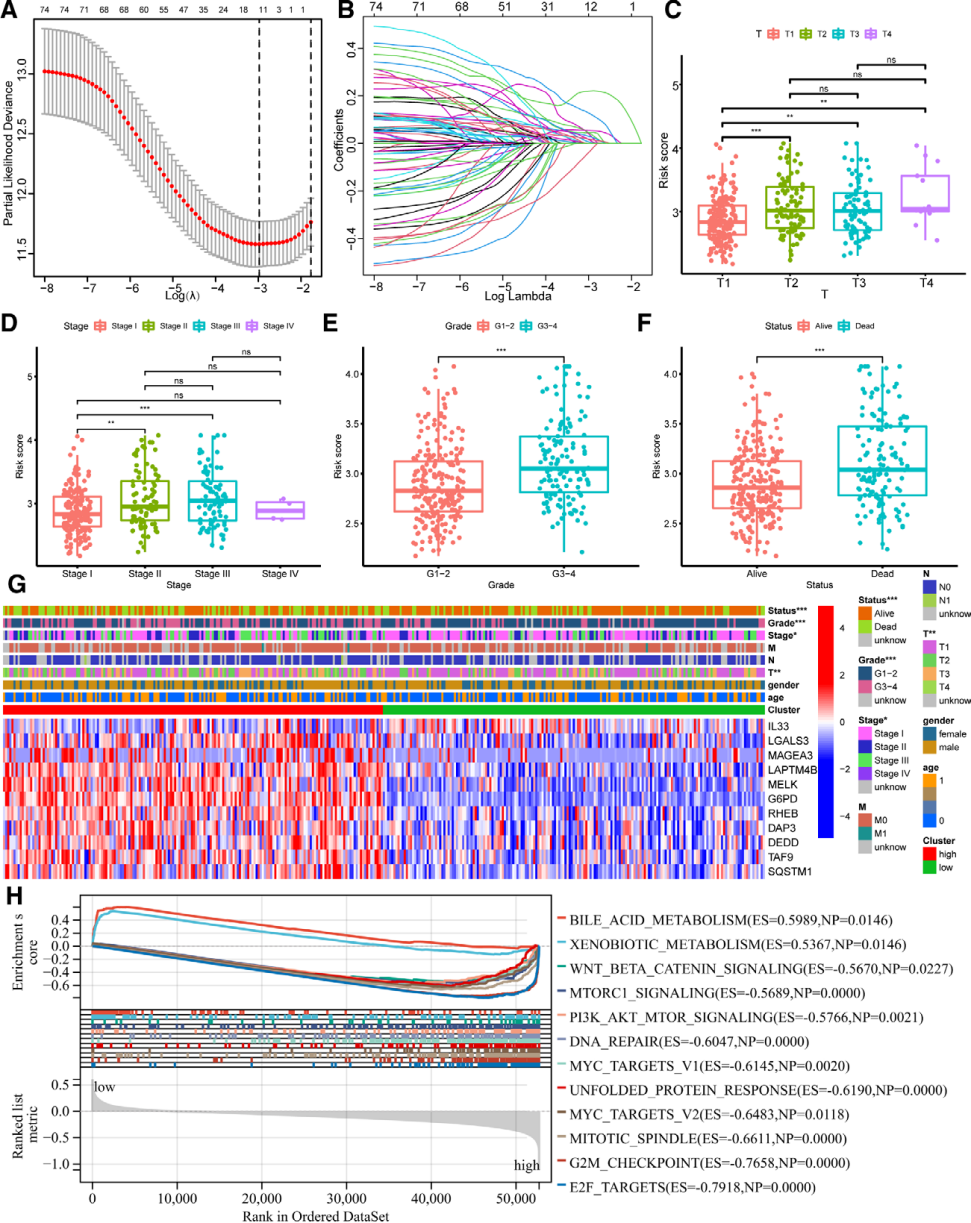
Construction of a prognostic gene signature for LIHC patients. (A) Selection of the 11 model genes. (B) Cross-validation of the constructed signature. Boxplots of the relationship between PCDscore and different T (C), clinical Stage (D), Grade (E), and survival status (F). *** Means *P* < .001; ** Means *P* < .01; * Means *P* < .05; ns Means not significant. (G) Heatmap of 11 model genes and clinical features. (H) GSEA analysis of top ten enrichment biological functions between 2 PCDscore groups in TCGA-LIHC cohort. GSEA = gene set enrichment analysis, LIHC = hepatocellular carcinoma.

We constructed an 11-gene signature (DAP3, DEDD, IL33, LGALS3, MAGEA3, MELK, TAF9, G6PD, RHEB, SQSTM1, LAPTM4B) using LASSO Cox regression analysis based on the prognostic PCD-related DEGs (as shown in Fig. 3A and B). Two of these genes were associated with apoptosis, 2 with ferroptosis, one with autophagy, and one with lysosome-dependent cell death. IL33 was found to play a role in both apoptosis and necroptosis, while SQSTM1 was found to be involved in autophagy, necroptosis, and lysosome-dependent cell death.

We calculated the PCDscore of each LIHC patient using our model, which was established by the formula: PCDscore = 0.046 × DAP3 + 0.037 × DEDD + (-0.034) × IL33 + 0.043 × LGALS3 + 0.029 × MAGEA3 + 0.055 × MELK + 0.006 × TAF9 + 0.221 × G6PD + 0.126 × RHEB + 0.015 × SQSTM1 + 0.018 × LAPTM4B. The 225 LIHC patients in the TCGA cohort were then divided into high-PCDscore and low-PCDscore groups based on the median PCDscore, which served as the training dataset. The PCDscore was significantly associated with various clinical features, including T (T1–T4), Grade (G1-2vs G3-4), clinical Stage (I–IV), and survival status (alive or dead) (Fig. 3C–F). Moreover, we compared the expression levels of the 11 model genes between the high and low groups (Fig. 3G) and found that all genes, except for IL33, were significantly upregulated in the high-PCDscore group.

We applied GSVA to further investigate the differences in biological processes between the subgroups classified by the gene signature. The low-PCDscore group was primarily enriched in metabolism-related pathways, including bile acid metabolism and xenobiotic metabolism (Fig. 3H). In contrast, the high-PCDscore group was mainly enriched in cancer- and immune-related pathways, such as WNT BETA CATANIN signaling, MTORC1 signaling, and PI3K AKT MTOR signaling (Fig. 3H). Detailed pathways are listed in Supplementary Table 4, http://links.lww.com/MD/K819.

### 3.3. Internal training and external validation of the prognostic PCD-related gene signature

**Figure 4.**
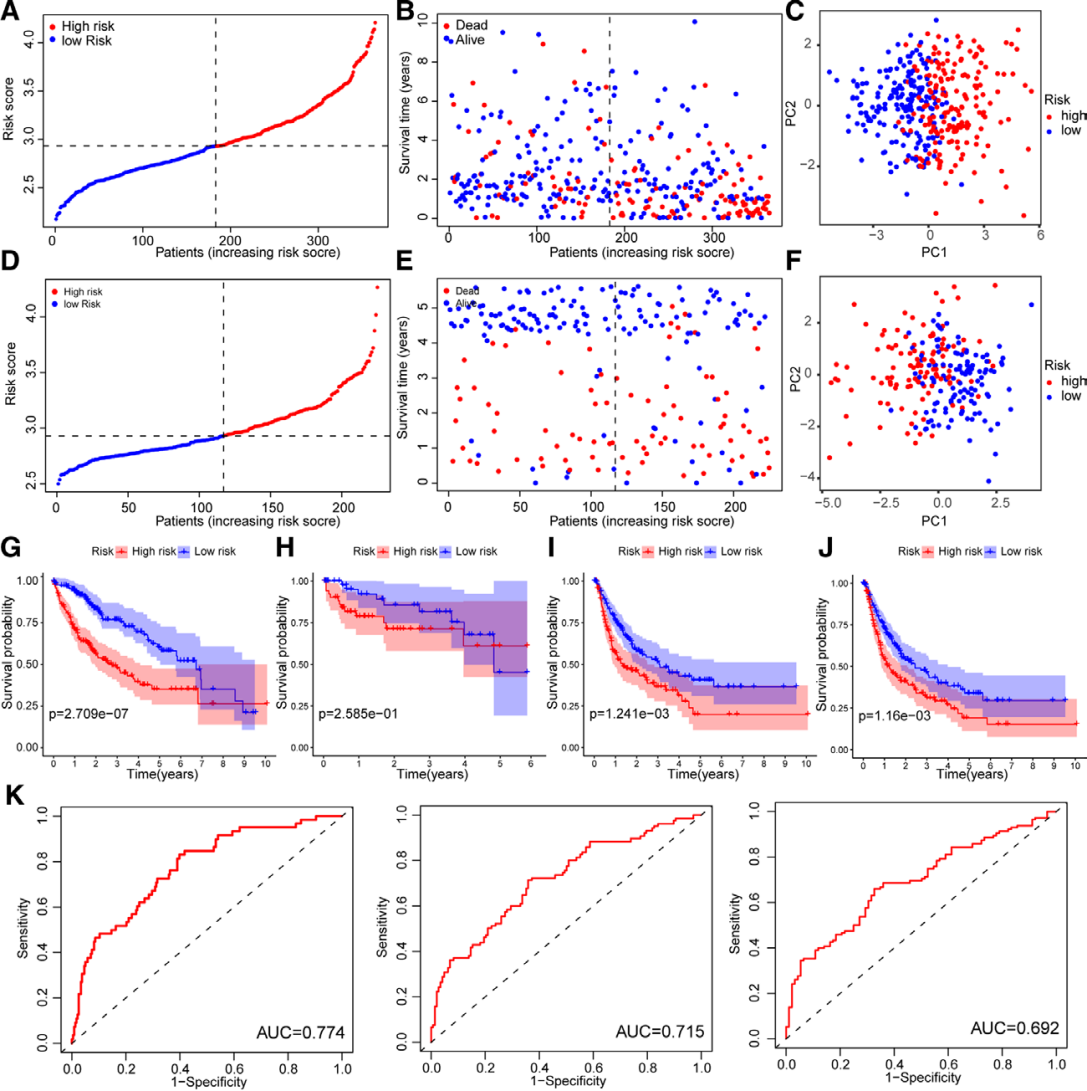
Internal training and external validation of the gene signature prediction model. (A-B) Distribution of PCDscore according to the survival status and time in TCGA-LIHC cohort. (C) Principal component analysis (PCA) plot based on the PCDscore in TCGA-LIHC cohort. (D-E) Distribution of PCDscore according to the survival status and time in GSE14520 cohort. (F) PCA plot based on the PCDscore in GSE14520 cohort. (G-H) Overall survival in the low- and high- PCDscore group patients in TCGA and GSE14520 cohorts. (I-J) Disease-free interval (DFI) and progression-free interval (PFI) in the low- and high- PCDscore group patients in TCGA-LIHC cohort. (K) ROC analysis of the 1-, 2-, and 3-yr survival rates for TCGA-LIHC patients. LIHC = hepatocellular carcinoma, ROC = receiver operating characteristic.

We proceeded to compare the OS between LIHC patients categorized by their PCDscore. Our findings indicated that patients with high PCDscore had significantly lower survival rates than those with low PCDscores (Fig. 4A and B). Furthermore, PCA showed that the classification based on PCDscore was accurate and reliable (Fig. 4C). A significant difference in OS time was observed between the high-PCDscore and low-PCDscore groups, with patients in the former exhibiting higher mortality rates (*P* < .05, Fig. 4G). To further validate the gene signature, we used the GSE14520 dataset as a validation cohort. Our findings indicated that patients with high PCDscore had significantly lower survival rates than those with low PCDscores (Fig. 4D and E). PCA analysis demonstrated satisfactory separation of the 2 groups (Fig. 4F). Consistent with the results of the training cohort, patients with a high PCDscore had a poor OS time (*P* < .05, Fig. 4H). Additionally, patients in the high-PCDscore group had worse DFI and PFI (*P* < .05, Fig. 4I and J). Furthermore, ROC analysis revealed that the 1-, 2-, and 3-year survival rates for LIHC were 0.774, 0.715, and 0.692, respectively (Fig. 4K).

### 3.4. Identification of subtypes using unsupervised clustering of the prognostic PCD-related gene signature

**Figure 5.**
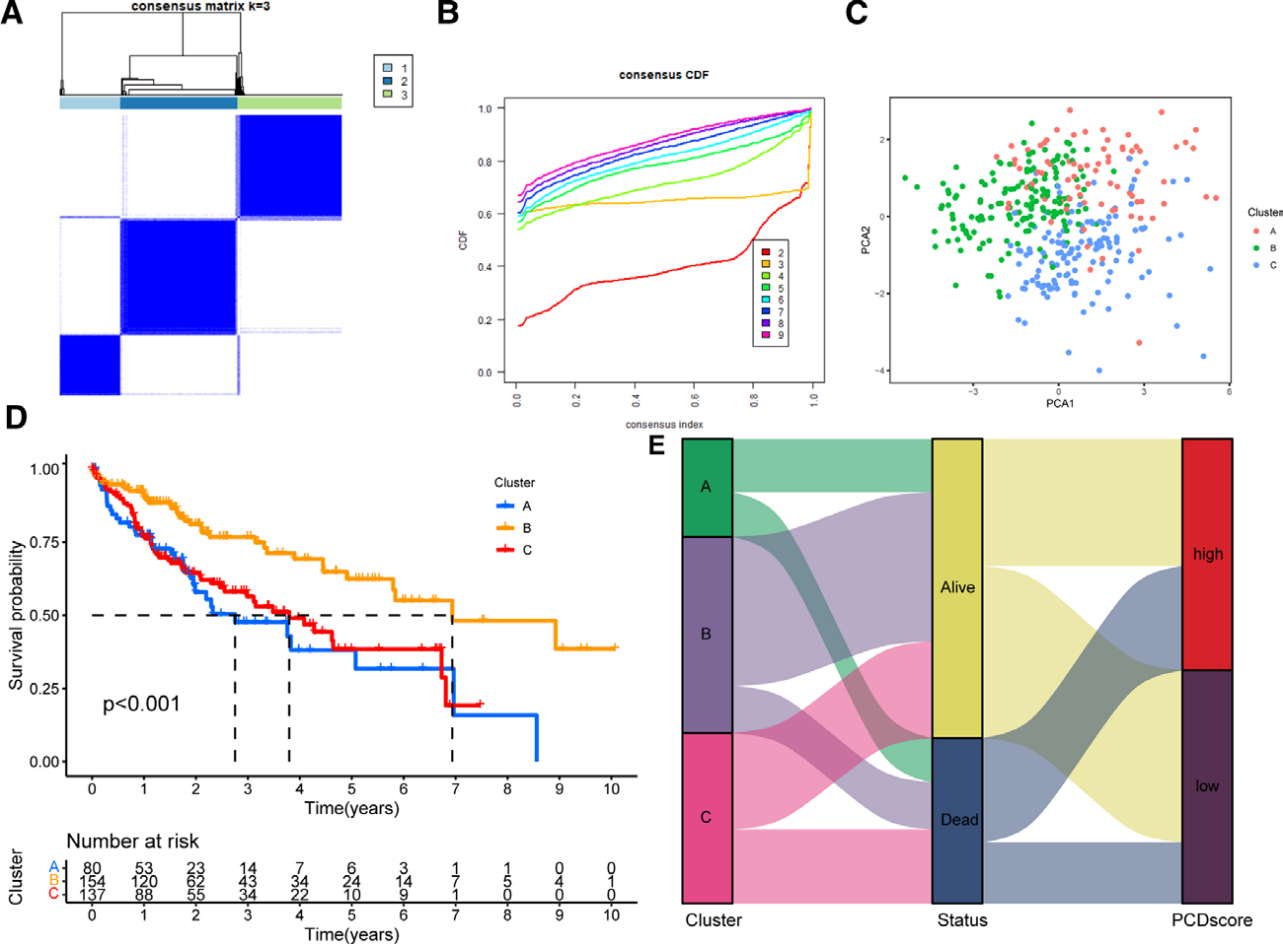
Unsupervised clustering of PCD related model genes. (A) TCGA-LIHC patients were grouped into 2 molecular clusters when k = 3, based on the programmed cell death model gene profile. (B) Empirical cumulative distribution function plot displaying consensus distributions for each k value (from 2 to 9). (C) Principal component analysis (PCA) plot based on the 11 model genes in TCGA-LIHC cohort. (D) Kaplan–Meier analysis of the prognosis of LIHC patients belonging to 3 different molecular clusters. (E) Alluvial diagram shows the interrelationship between molecular clusters, survival status, and PCDscore groups in LIHC patients. LIHC = hepatocellular carcinoma.

To identify potential subtypes of LIHC, we performed consensus clustering analysis using the 11 PCD-related model genes. Our results showed that the patients could be grouped into 3 distinct clusters, which we defined as Cluster A, B, and C, with the most significant differences among subgroups observed at k = 3 (Fig. 5A and B). The 3 clusters were well separated based on PCA analysis (Fig. 5C). There were significant differences in the OS time among the 3 clusters (*P* < .001, as shown in Fig. 5D). The favorable prognosis was associated with Cluster B, while Clusters A and C were associated with poor prognosis. Furthermore, the alluvial diagrams demonstrated the relationship between the 3 clusters and the PCDscore groups (Fig. 5E).

### 3.5. Developing and evaluating the nomogram survival model

**Figure 6.**
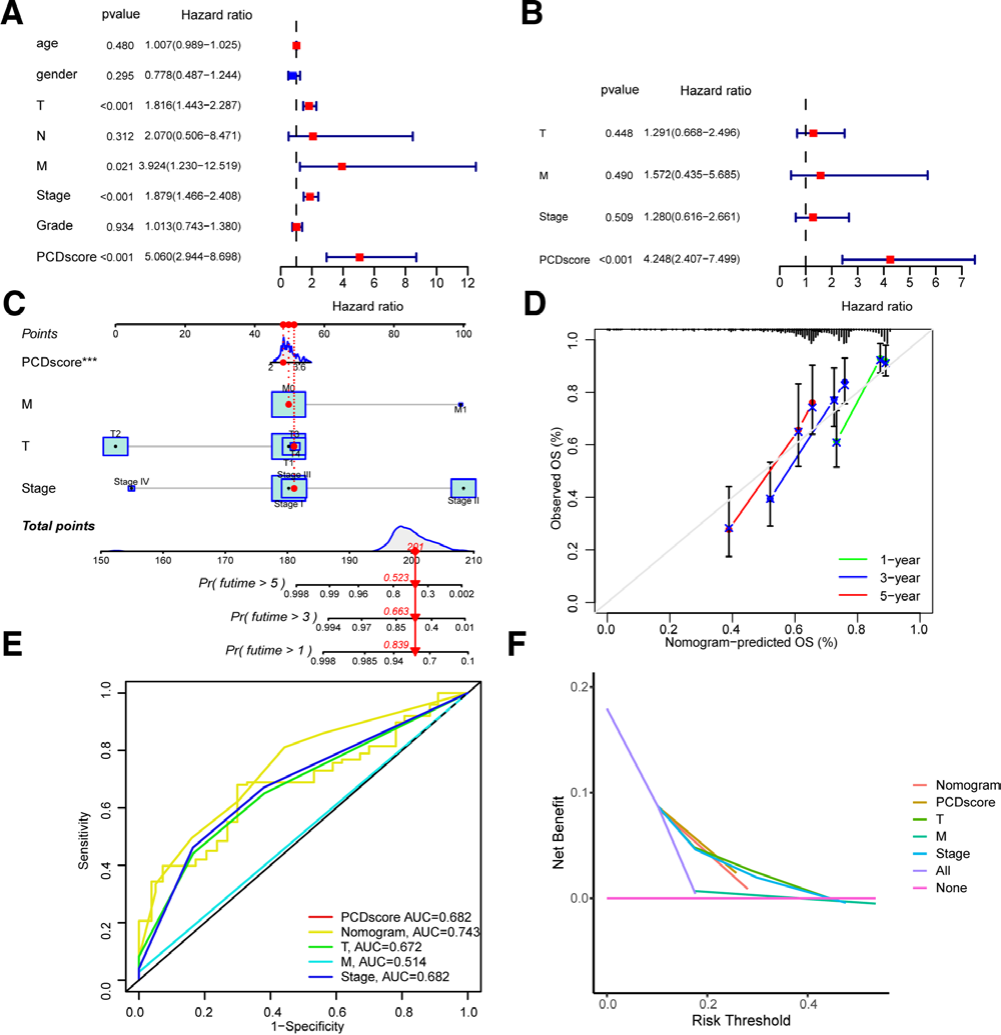
Establishment and assessment of the nomogram survival model. (A) Univariate analysis for the clinicopathologic characteristics and PCDscore in TCGA-LIHC cohort. (B) Multivariate analysis for the clinicopathologic characteristics and PCDscore in TCGA cohort. (C) A nomogram was established to predict the prognostic of LIHC patients. (D) Calibration plots showing the probability of 1-, 3-, and 5-yr OS in TCGA cohort. (E) Receiver operator characteristic (ROC) analysis of nomogram in TCGA-LIHC cohort. (F) Decision curve analysis (DCA) of nomogram predicting 1-, 3-, and 5-yr overall survival. LIHC = hepatocellular carcinoma, OS = overall survival.

To determine whether PCDscore could serve as an independent prognostic factor, we conducted both univariate and multivariate Cox regression analyses. Our results showed that PCDscore was identified as a risk factor in the univariate Cox regression analysis, with a hazard ratio of 5.060 and a 95% confidence interval (CI) of 2.944 to 8.698 (*P* < .001, Fig. 6A). Furthermore, after adjusting for other confounding factors, the multivariate analysis also confirmed that PCDscore remained an independent prognostic factor in LIHC patients, with an hazard ratio of 4.248 and a 95% CI of 2.407 to 7.499 (*P* < .001, Fig. 6B). We developed a nomogram model using T, M, stage, and PCDscore to estimate the 1-, 3-, and 5-year OS of LIHC patients in the TCGA cohort (Fig. 6C). The reliability of the model in predicting the 1-, 3-, and 5-year survival rates was demonstrated by the calibration curves (Fig. 6D), and the area under the curve values showed high accuracy in predicting the 5-year survival of LIHC patients (Fig. 6E). Furthermore, decision curve analysis showed that the nomogram model outperformed other predictors used in this study (Fig. 6F).

### 3.6. PCDscore gene signature to assess tumor immune characteristic and drug sensitivity

**Figure 7.**
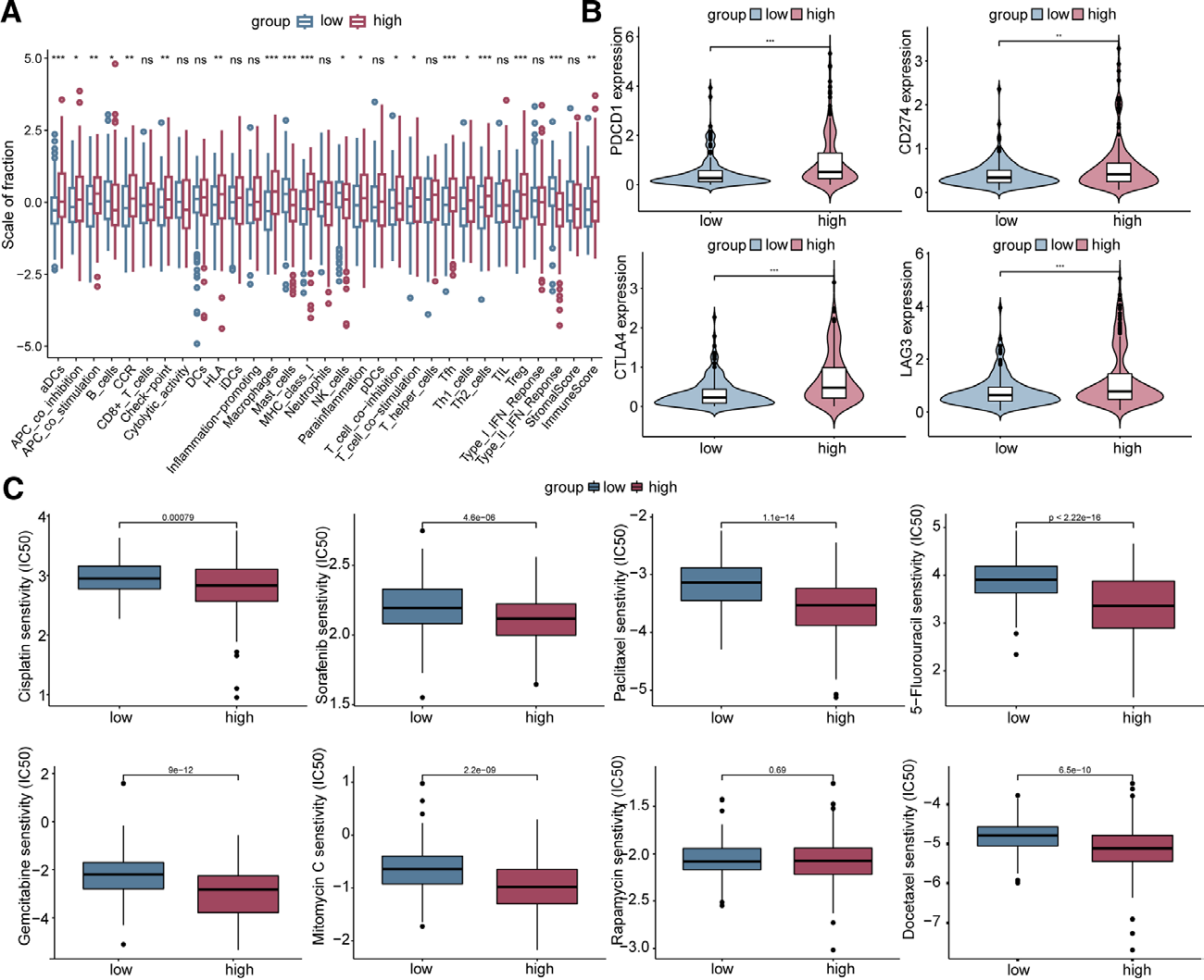
Immunological characterization and drug sensitivity analysis of high- and low-PCDscore groups. (A) The expression of immune function between different groups. (B) The expression levels of immune checkpoint genes between different groups. (C) The boxplot of sensitivity of common chemotherapy drugs in different groups. ns, not significant, *P < .05, **P < .01, ***P < .001.

The ssGSEA analysis revealed differential immune cell infiltration levels, immune functions, and immune scores between the high and low PCDscore groups (Fig. 7A). The high PCDscore group was enriched with immune functions related to APC co-stimulation, APC co-inhibition, check point, HLA, MHC class I, and type II IFN response (Fig. 7A). In addition, immune checkpoint genes such as PDCD1, CTLA4, and LAG3 were significantly upregulated in the high PCDscore group (Fig. 7B). Moreover, high PCDscore patients exhibited lower the half-maximum inhibitory concentration values for most chemotherapeutic agents (Fig. 7C), indicating that PCDscore may serve as a potential predictor of chemosensitivity.

## 4. Discussion

This study represents the first comprehensive analysis of twelve diverse PCD patterns in LIHC, leading to the construction of a highly effective PCD-related gene signature in the TCGA cohort, with validation in the external cohort (GSE14520). A nomogram was developed that incorporated clinical characteristics and PCDscore, which demonstrated excellent performance. We also investigated the correlation between PCDscore and tumor microenvironment, immune checkpoint related genes, and drug sensitivity. PCD is regulated through intricate mechanisms that involve various processes. A mounting body of evidence suggests that PCD plays a crucial role in biological processes and has been demonstrated to be linked to the development and advancement of malignant tumors for many years.^[25]^

Predictive models utilizing gene expression profiling serve as robust prognostic indicators of patient outcomes. Previous studies have predicted liver cancer prognosis by constructing signatures such as microRNA signatures and lncRNA signatures.^[7,34]^ We identified 75 strong prognostic PCD-related genes, which participate in multiple critical carcinogenic biological pathways. Based on these genes, we developed a gene signature consisting of 11 PCD-related genes (DAP3, DEDD, IL33, LGALS3, MAGEA3, MELK, TAF9, G6PD, RHEB, SQSTM1, LAPTM4B) and observed that it was capable of predicting the OS, DFI, and PFI in patients with LIHC. Most of these genes, except for DEDD and IL33, had also been included in different prognostic models of LIHC in previous studies.^[35–43]^ The protein DEDD (death effector domain-containing protein) has been implicated in numerous physiological processes, including apoptosis.^[44]^ Recent studies have suggested that DEDD could potentially serve as a valuable prognostic marker and therapeutic target for the treatment of cancer metastasis.^[45,46]^ In a noninjurious model of liver resection, IL33 (Interleukin-33) has been shown to promote regeneration and decrease the likelihood of post-hepatectomy liver failure.^[47]^ In our study, we observed an upregulation of IL-33 expression in patients with a low PCDscore. Meanwhile, the expression levels of the other genes were found to be increased in patients with a high PCDscore, consistent with the results reported in previous studies. For example, MELK has been shown to be upregulated in liver cancer and may possess oncogenic properties in LIHC, and it is also associated with immune cell infiltration and poor prognosis.^[48]^ RHEB overexpression is correlated with metastasis and an unfavorable prognosis in hepatocellular carcinoma.^[49]^ Studies have shown that LAPTM4B gene overexpression is correlated with the malignant phenotypes of hepatocellular carcinoma.^[50]^ Moving forward, it will be necessary to conduct additional experiments for an in-depth exploration of the functions and mechanisms of these genes in patients with LIHC or patients with LIHC who had a history of Hepatitis C Virus.^[51]^

In order to investigate the potential mechanisms underlying the significant difference in prognosis between the high-PCDscore and low-PCDscore groups, we conducted GSVA and GSEA analyses to explore the biological processes associated with each group. Our results showed that the low-PCDscore group was significantly enriched in metabolism-related pathways, including bile acid metabolism and xenobiotic metabolism. Cancer-related signaling pathways, such as WNT-BETA-CATANIN, MTORC1, G2M checkpoint, and PI3K-AKT-MTOR signaling were significantly enriched in the high-PCDscore group, which were demostrated to be associated with anti-tumor immune response.^[52–55]^ Tumor cells can evade immune surveillance and resist drug therapy due to the supportive tumor microenvironment.^[56,57]^ In theory, the high-PCDscore group may exhibit decreased expression levels of immune checkpoints and reduced infiltration of anti-tumor immune cells, indicating an overall impairment of immune function.^[58,59]^ However, the activity of PCD is positively associated with anti-inflammatory activity, which may provide a plausible explanation for our findings. In the high-PCDscore group, we observed upregulation of PCD gene expression. Additionally, ssGSEA analysis demonstrated significant differences in immune cell infiltration between the 2 groups, with the high-PCDscore group showing a higher immune score than the low-PCDscore group. The results indicate that the PCD-related gene signature exhibits distinct characteristics in both biological pathways and immune cell infiltration within the tumor microenvironment (TME).

With the deeper investigation of tumor immunology and molecular biology, immune checkpoint blockers (ICBs) have emerged as a promising approach for cancer treatment, including LIHC.^[60–62]^ The investigation of immune checkpoint inhibitors (ICIs) targeting CTLA-4, PD-1, and PD-L1 is rapidly advancing, with clinical trials demonstrating their effectiveness and safety.^[63,64]^ In recent years, an increasing number of studies have focused on the application of ICIs in liver cancer.^[65,66]^ The high-PCDscore group exhibited significantly higher expression of immune checkpoint genes (PD-1, PDL-1, CTLA4, and LAG3) compared to the low-PCDscore group in our study, implying a possible sensitivity to ICIs targeting these checkpoint molecules in these patients. As the understanding of adjuvant chemotherapy deepens, selecting the appropriate chemotherapy drugs can improve the prognosis of patients with liver cancer.^[67]^ Furthermore, we assessed the association between the PCD-related gene signature and the sensitivity to conventional chemotherapy drugs used in LIHC treatment. Our findings demonstrated that the high-PCDscore group exhibited greater sensitivity to chemotherapy agents such as cisplatin, paclitaxel, and docetaxel, highlighting the potential of the PCD-related gene signature in guiding personalized treatment strategies and providing medication recommendations for LIHC patients.

The study has some limitations despite the PCD-related gene signature demonstrating excellent prognostic performance in both training and validation cohorts and the ability to predict anti-tumor therapy response in LIHC. Firstly, all expression data was sourced from public databases, and the findings require confirmation using new cohorts and fresh specimens. Additionally, the potential to predict anti-tumor therapy response was evaluated indirectly, and further research are required to validate our conclusion.

## 5. Conclusions

Conclusively, the PCD-related gene signature proposed in this study is a reliable prognostic indicator for postoperative LIHC patients, and could significantly impact clinical outcome assessment. These findings underscore the clinical relevance of the PCD-related gene signature and offer a novel approach for tailoring individualized therapy for LIHC patients.

## Author contributions

**Conceptualization:** Xiaobo Wang.

**Data curation:** Youlin Yu, Yanglieguang Lou, Jinlong Zhu.

**Formal analysis:** Youlin Yu, Jinlong Zhu.

**Investigation:** Yanglieguang Lou.

**Methodology:** Youlin Yu, Yanglieguang Lou, Jinlong Zhu.

**Project administration:** Xiaobo Wang.

**Supervision:** Xiaobo Wang.

**Validation:** Yanglieguang Lou.

**Visualization:** Youlin Yu, Jinlong Zhu.

**Writing – original draft:** Youlin Yu, Yanglieguang Lou, Jinlong Zhu.

**Writing – review & editing:** Xiaobo Wang.

## Supplementary Material








